# Effect of preterm infant behavioral assessment education on maternal stress and attachment: comparison of booklet-based and face-to-face education

**DOI:** 10.1186/s12884-026-09712-y

**Published:** 2026-07-27

**Authors:** Mahni Rahkar Farshi, Nastaran Arabi, Mahnaz Jabraeili

**Affiliations:** 1 Pediatric Department, Faculty of Nursing and Midwifery, Tabriz University of Medical Sciences, Tabriz, Iran; 2 Neonatal Intensive Care Department, Tabriz Al-Zahra Hospital, Tabriz University of Medical Sciences, Tabriz, Iran; 3 Pediatric Department, Faculty of Nursing and Midwifery, Tabriz University of Medical Sciences, South Shariati, Tabriz, Iran

**Keywords:** Preterm infant, Maternal stress, Attachment, Behavioral assessment, Nursing education

## Abstract

**Background:**

Mothers of preterm infants hospitalized in neonatal intensive care units (NICUs) often experience high stress levels and challenges in developing early mother–infant attachment.

Educational interventions based on the Assessment of Preterm Infants’ Behavior (APIB) may improve mothers’ understanding of infant behavioral cues and support maternal psychological adaptation in NICU settings. This study aimed to evaluate the effect of booklet-based and face-to-face APIB-based education on stress and attachment among mothers of preterm infants.

**Methods:**

The present quasi-experimental study was conducted between February 2023 and January 2024 in the NICU of a referral hospital. This study included 90 mothers of preterm infants assigned sequentially to booklet-based education (*n =* 30), face-to-face education (*n =* 30), or routine care (*n =* 30). Maternal stress and attachment were assessed before and after intervention using the Parental Stressor Scale and Maternal Attachment Inventory. Data were analyzed using ANCOVA and adjusted pairwise comparisons.

**Findings:**

Maternal stress decreased significantly in all groups (*p* < 0.001). Adjusted comparisons showed significantly lower stress in the booklet-based education group (mean difference = − 6.09, *p* = 0.004) and face-to-face education group (mean difference = − 4.16, *p* = 0.040) versus control, with no difference between intervention groups (*p* = 0.338). Maternal attachment increased significantly within both intervention groups, but no significant between-group differences were observed after adjustment (*F =* 0.94, *p* = 0.394).

**Conclusion:**

APIB-based education may provide benefits beyond routine NICU support by improving maternal interpretation of infant behavioral cues. Longer follow-up may be required to detect changes in attachment. APIB-based parental education may be a feasible strategy for reducing maternal stress in NICUs and could be incorporated into family-centered neonatal care to support mothers of preterm infants.

## Background

Preterm birth rates and mortality vary significantly between and within countries, with low- and middle-income countries bearing a disproportionately high burden [1, 2]. Recent evidence from Iran indicates that approximately 10.48% of all births are preterm. In a prospective birth cohort study, 8.75% of infants were classified as late preterm, 1.26% as moderate preterm, and 0.52% as very or extremely preterm [3].

Mothers of preterm infants experience significantly higher levels of stress [4, 5]. The neonatal intensive care unit (NICU) environment, characterized by technological equipment and limited physical contact, further intensifies maternal stress [6]. Evidence suggests that elevated maternal stress may persist beyond hospitalization and negatively impact mother-infant interactions [7]. Challenges in understanding preterm infant behavior also contribute to maternal stress. Preterm infants display distinct behavioral and neurodevelopmental characteristics compared with term infants, including less stable autonomic regulation, altered sleep–wake states, and subtle interactional cues [8]. These immature behavioral patterns can make it difficult for mothers to accurately interpret their infant’s signals [9]. Misinterpretation of normal preterm behaviors, such as gaze aversion, motor disorganization, or physiological instability, may be perceived by mothers as signs of rejection or illness, thereby increasing maternal anxiety and stress [10, 11]. Within this framework, caregiver understanding of infant behavior plays a crucial role in promoting positive early interactions [12].

Fostering mothers' sensitivity to recognize their infants' cues can facilitate the formation and continuation of attachment [13]. Maternal–infant attachment is a fundamental predictor of infant socio-emotional and cognitive development [14], yet preterm birth and subsequent NICU hospitalization often disrupt early bonding by limiting physical contact and shared interaction opportunities [15, 16]. Maternal stress from separation in the NICU can impair sensitive caregiving and attachment behaviors like eye contact and affective attunement [17, 18]. Research connects heightened maternal stress in the NICU to difficulties in secure attachment, as interpreting infant cues becomes challenging and undermines early relational patterns [19, 20].

Educational and supportive interventions for mothers of preterm infants have been associated with improved psychological and relational outcomes. Sensitivity-based and parent-focused programs have demonstrated increases in maternal attachment, parenting confidence [21, 22], and self-efficacy [22, 23], alongside reductions in parental stress [21, 24]. Systematic [25] and scoping [26] reviews further indicate that NICU-based educational and psychosocial interventions contribute to decreased anxiety and enhanced caregiving readiness.

Various modes of educational delivery have been used to support parents of infants, including direct instruction, written educational materials, technology-based interventions, and combined methods [27]. Face-to-face education enables interactive dialogue, immediate clarification, and tailored guidance, which may enhance parental understanding and engagement [27, 28]. In contrast, written educational methods provide accessible, low-cost, and self-paced learning opportunities that allow repeated review of information [29, 30]. Emerging evidence suggests that the method of educational delivery may influence parental knowledge acquisition, confidence, and psychological outcomes; however, comparative findings across different delivery formats remain inconsistent [27].

Therefore, evaluating educational approaches is crucial for identifying the most effective strategies to support mothers of preterm infants. Despite the expansion of parent-focused NICU interventions, research on programs specifically addressing structured assessment and interpretation of preterm infant behavior remains limited. This study compared the effectiveness of booklet-based and face-to-face preterm infant behavioral assessment education on stress and attachment among mothers of preterm infants.

## Methods

### Study design

This three-arm study used a quasi-experimental design because participant sharing of educational content could not be fully controlled. Educational interventions were therefore delivered sequentially to each group. The study design and reporting followed Transparent Reporting of Evaluations with Nonrandomized Designs (TREND) guidelines [31].

### Setting and participants

The study was conducted between February 2023 and January 2024 in the NICU of referral hospital affiliated with Tabriz University of Medical Sciences. This hospital is the only referral center for preterm infants in northwest Iran. Convenience sampling was used to recruit mothers based on predefined inclusion criteria. Mothers had to be first-time mothers of preterm infants born between 28 and 34 weeks of gestation and admitted to the NICU, and were capable of reading and writing. Exclusion criteria included irregular maternal visits, known physical or psychological illness, infant discharge, or death during the study, clinical deterioration requiring resuscitation or mechanical ventilation, infants conceived after prolonged infertility, congenital anomalies, or recent major family stressors such as bereavement, serious illness, or parental separation.

Baseline demographic and clinical characteristics of mothers and infants were collected using a structured questionnaire. Maternal variables included age, educational level, employment status, and mode of delivery. Infant clinical characteristics comprised gestational age, birth weight, Apgar scores at 1 and 5 min, sex, and diagnosis.

### Intervention

This study included two intervention groups and one control group. In addition to routine NICU education, mothers in the intervention groups received identical educational content delivered through two different modalities: face-to-face instruction and booklet-based education. The control group received routine unit education only.

#### Development of the APIB-based educational content

The educational content was developed based on the Assessment of Preterm Infants’ Behavior (APIB) framework introduced by Als [8]. The content addressed interpretation of preterm infant behavior within five subsystems: autonomic, motor, state organization, attention–interaction, and self-regulation. Additional components included definition and physical characteristics of preterm infants and orientation to the NICU-specific care and equipment (Table 1).

**Table 1:** Content of the APIB-based educational intervention in the intervention groups: face-to-face and booklet-based education

Topic	Content overview^*^
Definition of preterm infant	Preterm birth (30–34 weeks), physical characteristics, and developmental immaturity of organ systems. Key reflexes and general behaviors introduced
Care in NICU	Orientation to specialized care, NICU equipment, and rationale for interventions. Emphasis on maternal observation and safe handling
Autonomic system behaviors	Identification of stress and stability cues in respiration, color, and temperature regulation. Common digestive system symptoms: bowel movements, burping, and hiccups. Dynamic interactions with other subsystems highlighted
Motor systems behaviors	Positions and posture, muscle tone, and spontaneous movements. Dynamic interactions with other subsystems highlighted
State organization	Recognition of sleep–wake states, alertness, gaze patterns, and social responsiveness
Attention–interaction	Identification of alertness and engagement cues, and gaze patterns. Guidance on timing and quality of maternal interactions to support stability
Self-regulation system	Observation of self-soothing and equilibrium-restoring behaviors. Maternal strategies to support infant regulation and reduce stress

^*^Each subsystem emphasizes inter-system interactions, stress vs. stability cues, and environmental influences

To establish content validity, the material was reviewed by an expert panel of ten faculty members specializing in neonatal nursing and neonatology at Tabriz University of Medical Sciences. Modifications were incorporated based on their feedback prior to implementation. All educational procedures across study groups were delivered by a trained research nurse experienced in neonatal care and fully trained in the educational content.

Maternal comprehension was assessed immediately following completion of the educational content. For each behavioral subsystem, five standardized questions were asked. When incorrect or incomplete responses were identified, targeted clarification was provided to ensure adequate understanding before completion of the session. Follow-up procedures were standardized across groups, and contact frequency and duration kept similar to minimize differential attention effects.

#### Face-to-face education group

In the face-to-face education group, delivery of the educational content began 2–4 days after infant admission to the NICU, contingent upon maternal readiness. Education was conducted in small groups of 3–5 mothers in a designated classroom within the unit. The educational content was delivered over three consecutive days in three sessions of 45 min each (total: 135 min). Instruction was provided using simple, non-technical language and supported by printed visual materials illustrating behavioral cues and subsystem indicators. After the final session, a structured 10-min individual question-and-answer session was conducted to verify comprehension and address remaining questions.

#### Booklet-based education group

In the booklet-based education group, the same educational content was provided 2–4 days after infant admission, contingent upon maternal readiness. The material was presented in a booklet containing photographs and simplified explanations identical in scope and structure to that delivered in the face-to-face education group. Mothers were given three days for independent review of the material. After this period, a structured 10-min individual question-and-answer session was conducted to clarify ambiguities and ensure adequate understanding of the educational content. The research nurse performed follow-up checks during mothers’ presence in the unit to encourage engagement with the material.

#### Control group

Mothers in the control group received routine NICU education 2–4 days after infant admission upon maternal readiness. This education included instruction on kangaroo mother care, infant massage, and breastfeeding techniques and was delivered over three consecutive days. After the routine education, a structured 10-min individual question-and-answer session was held to address questions and confirm understanding of the routine education.

### Objectives

The primary objective of this study was to evaluate the effect of APIB-based behavioral assessment education, delivered either face-to-face or through a booklet in addition to routine care, on maternal stress among mothers of preterm infants admitted to the NICU. The secondary objective was to examine the impact of this intervention on maternal attachment and to compare outcomes across the three study groups.

### Outcome

#### Outcome assessment procedure

The study outcomes were maternal stress and maternal attachment. Assessments were conducted at two time points: pre-intervention (pre-test) and post-intervention (post-test). In the intervention groups, pre-intervention assessment was performed 2–4 days after infant admission and prior to initiation of the educational content, that is, before the first face-to-face session or before receiving the booklet. In the control group, pre-intervention assessment was conducted 2–4 days after infant admission to the NICU, to ensure consistency with the timing used in the intervention groups. Post-intervention assessment was conducted 48 h after completion of the final educational session in the face-to-face education group, 48 h after completion of the three-day booklet review period in the booklet-based education group, and 48 h after completion of the three-day routine education in the control group. Outcome assessment at both time points was performed by a research assistant, who was blinded to group assignment and was not involved in delivering the intervention. All questionnaires were completed by mothers in a quiet room within the NICU under the supervision of this research assistant. 

#### Maternal stress

Maternal stress was measured using the Parental Stress Scale (PSS) developed by Berry and Jones [32]. The scale consists of 18 items rated on a 5-point Likert scale (strongly disagree: 1, disagree: 2, no opinion: 3, agree: 4, and strongly agree: 5). Items 1, 2, 5, 6, 7, 8, 17, and 18 are reverse scored. Total scores range from 18 to 90, with higher scores indicating greater parental stress [32]. The original developers reported internal consistency of 0.83 and test–retest reliability of 0.80 [32]. The PSS is a widely used instrument with demonstrated validity and satisfactory reliability across different populations. Recent evidence from a systematic review and meta-analysis supports its psychometric robustness for assessing parental stress [33]. In our study, content validity of the scale was assessed by a panel of 10 experts. All items demonstrated a content validity ratio (CVR) greater than 0.80, and the overall content validity index (CVI) was 0.94. The reliability was evaluated using Cronbach’s alpha, with a coefficient of 0.79.

#### Maternal attachment

Maternal attachment was assessed using the Maternal Attachment Inventory (MAI) developed by Muller in 1994 and later revised to a final 26-item version. The items are rated on a 4-point Likert scale (almost always: 4, always: 3, sometimes: 2, and almost never: 1). Total scores range from 26 to 104, with higher scores reflecting stronger maternal attachment [34]. In our study, content validity of MAI was assessed by a panel of 10 experts. All items showed good content validity (CVR > 0.82; overall CVI = 0.89) and high reliability (Cronbach's alpha = 0.87).

### Sample size

Sample size was determined based on maternal stress as the primary outcome. The mean within-subject difference estimated from pilot data from 10 mothers yielded an effect size of 0.7. Assuming a two-sided alpha of 0.05 and 80% power, using G*Power 3.1.9.7 software [35], the required sample size was 28 participants for each group. Considering a potential 10% attrition rate, a total of 30 participants were included.

### Assignment method

**Fig. 1:**
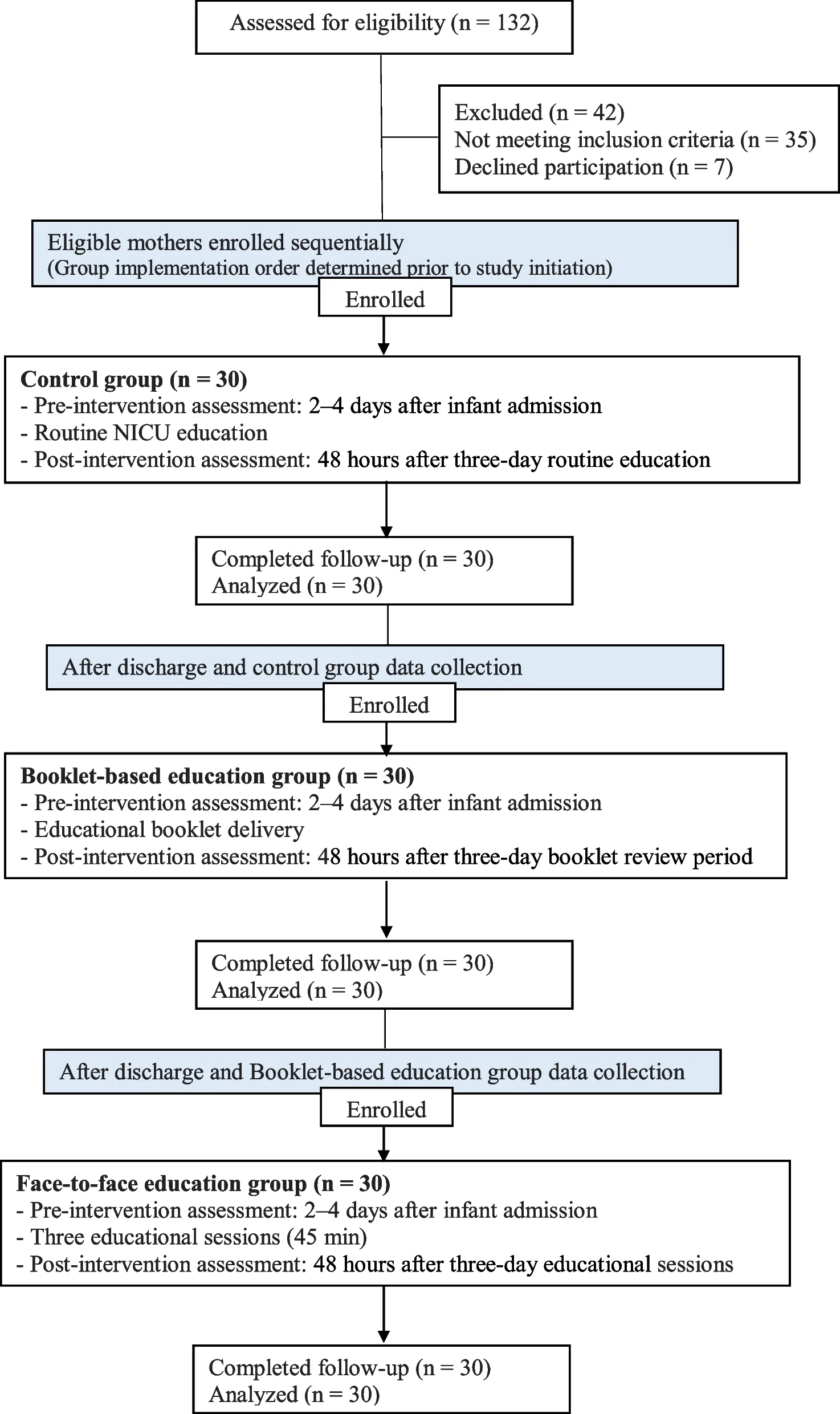
Flow diagram of participant recruitment. This diagram presents enrollment, assignment, follow-up, and analysis of study participants

Given the possibility of sharing information between the three study groups, the educational interventions for each group were carried out sequentially in this study. Specifically, after the intervention for one group was completed, and after its data were collected and the mothers were discharged, mothers in the next group were enrolled in the study. The order of intervention selection for the groups was randomized. Accordingly, the control group was enrolled first, followed by the booklet-based education group and subsequently the face-to-face education group (Fig. 1).

Due to the lack of randomization and the quasi-experimental nature of the study, efforts were made to ensure that the groups were comparable in terms of maternal age, education level, infant age and weight, and other identified variables in order to minimize potential confounding.

### Blinding

Due to the nature of the educational interventions, participants and the intervention provider were not blinded to group assignment. However, the outcome assessor was blinded to the group assignment.

### Statistical methods

Data were analyzed using IBM SPSS Statistics version 26. Normality of continuous variables was assessed with the Shapiro–Wilk test. Descriptive statistics are presented as mean and standard deviation (SD) for continuous variables and frequencies and percentages for categorical variables. Mothers were considered the unit of analysis for all outcomes. Baseline comparisons of demographic and clinical characteristics between the three study groups were performed using Chi-square and Fisher’s exact tests for categorical variables and one-way analysis of variance (ANOVA) for continuous variables. Intervention effects on maternal stress and attachment scores were examined using paired t-tests for within-group comparisons and one-way ANOVA for between-group comparisons. Additionally, analysis of covariance (ANCOVA) was conducted to compare post-intervention outcomes while adjusting for pre-intervention values of the corresponding variables, in order to obtain adjusted estimates of intervention effects. Where ANOVA indicated significant differences, post-hoc pairwise comparisons were conducted. A two-sided *p*-value < 0.05 was considered statistically significant.

## Results

A total of 90 mothers with preterm infants participated in the study and were assigned to the booklet-based education group (*n =* 30), face-to-face education group (*n =* 30), and control group (*n =* 30). No statistically significant differences were observed among the three groups regarding demographic and clinical characteristics, indicating comparability between groups prior to the intervention (Table 2).

**Table 2:** Demographic and clinical characteristics of mothers and their infants across study groups

		Booklet-based education group (***n =*** 30)	**Face-to-face education group (*n =* 30)**	Control group (***n =*** 30)		
**Continuous variables**		Mean (SD)	Mean (SD)	Mean (SD)	Test	*p*-value^•^
Maternal age (years)		30.76 (7.82)	31.03 (7.31)	30.70 (7.42)	*F =* 0.01	0.981
Gestational age (weeks)		32 (2.94)	31.73 (4.04)	33.37 (3.32)	*F =* 1.81	0.169
Infant weight (g)		1900.39 (573.00)	2291.80 (640.01)	2005.86 (665.92)	*F =* 0.61	0.544
Apgar score at 1 min		6.65 (1.86)	6.20 (2.40)	6.78 (2.14)	*F =* 0.62	0.539
Apgar score at 5 min		8.27 (1.04)	7.97 (1.44)	8.20 (1.49)	*F =* 0.42	0.658
**Categorical variables**		n (%)	n (%)	n (%)	Test	*p*-value^••^
Maternal education	Primary/secondary school	13 (43.33)	10 (33.33)	10 (33.33)	χ^2^ = 7.26	0.529
	Diploma	7 (23.33)	14 (46.67)	11 (36.67)		
	University	10 (33.34)	6 (20.00)	9 (30.00)		
Maternal employment status	Housewife	26 (86.66)	30 (100)	27 (90)	-	0.078^†^
	Employed	4 (13.33)	0 (0.00)	3 (10.00)		
Mode of delivery	NVD	7 (23.34)	8 (26.66)	2 (6.67)	-	0.067^†^
	C/S	23 (76.67)	22 (73.34)	28 (93.33)		
Infant sex	Female	14 (46.67)	18 (60.00)	12 (40.00)	χ^2^ = 4.77	0.268
	Male	16 (53.33)	12 (40.00)	18 (60.00)		
Infant diagnosis	RDS	28 (93.33)	23 (76.66)	26 (86.67)	-	0.212^†^
	TTN	2 (6.67)	7 (23.34)	4 (13.33)		

*Abbreviations*: *SD* Standard deviation, *n* number, *g* gram, *min* minutes, *NVD* Normal vaginal delivery, *C/S* Caesarean section, *RDS* Respiratory distress syndrome, *TTN* Transient tachypnoea of the newborn

•Continuous variables were compared using One-way ANOVA

••Categorical variables were compared using Chi-square test

^†^Corresponds to Fisher’s exact test that was used for categorical variables where expected cell counts were < 5

Maternal stress scores decreased significantly following the intervention in all three groups (all *p* < 0.001). However, post-intervention stress levels differed significantly between groups (*F =* 4.88, *p* = 0.010), with lower stress scores observed in both intervention groups compared with the control group (Table 3). Pairwise comparisons adjusted for pre-intervention stress scores showed significantly lower post-intervention stress in the booklet-based education group compared with the control group (adjusted mean difference = − 6.09, *p* = 0.004), and in the face-to-face education group compared with the control group (adjusted mean difference = − 4.16, *p* = 0.040). No significant difference was found between the two intervention groups (*p* = 0.338). ANCOVA indicated a significant overall intervention effect on maternal stress after adjustment for pre-intervention values (*F =* 4.68, *p* = 0.012, partial η^2^ = 0.096) (Table 4).

**Table 3:** Changes in maternal stress and attachment scores before and after the intervention across study groups

Maternal stress (Score range: 18–90)	Group	Pre-intervention Mean (SD)	Post-intervention Mean (SD)	Within-group comparison^•^
	Booklet-based education	50.03 (6.01)	33.63 (7.71)	t = 9.71, *p* < 0.001
	Face-to-face education	50.40 (9.18)	35.59 (7.01)	t = 7.46, *p* < 0.001
	Control	51.20 (6.92)	39.83 (8.80)	t = 5.92, *p* < 0.001
	Between-group comparison^••^	*F =* 0.18, *p* = 0.829	*F =* 4.88, *p* = 0.010	
Maternal attachment (Score range: 26–104)	Group	Pre-intervention Mean (SD)	Post-intervention Mean (SD)	Within-group comparison^•^
	Booklet-based education	50.60 (6.45)	54.36 (3.45)	t = −3.12, *p* = 0.004
	Face-to-face education	51.37 (5.48)	54.87 (3.09)	t = −2.82, *p* = 0.008
	Control	51.90 (3.16)	53.60 (4.25)	t = −1.69, *p* = 0.101
	Between-group comparison^••^	*F =* 0.47, *p* = 0.627	*F =* 0.96, *p* = 0.384

**Table 4:** Pairwise comparison of post-intervention maternal stress and attachment scores across study groups

	**Comparison**	**Adjusted Mean Difference**	**95% CI**	*p***-value**^•^	**ANCOVA**^••^ (F, p, η^2^)
Maternal stress	Booklet-based education vs Face-to-face education	−1.92	−5.90 to 2.05	0.338	*F =* 4.68 *p* = 0.012 Partial η^2^ = 0.096
	Booklet-based education vs Control	−6.09	−10.13 to −2.04	0.004	
	Face-to-face education vs Control	−4.16	−8.14 to −0.18	0.040	
Maternal attachment	Booklet-based education vs Face-to-face education	−0.51	−2.36 to 1.32	0.578	*F =* 0.94 *p* = 0.394 Partial η^2^ = 0.021
	Booklet-based education vs Control	0.75	−1.13 to 2.63	0.430	
	Face-to-face education vs Control	1.26	−0.57 to 3.11	0.175	

*Abbreviations*: *CI* Confidence interval, *ANCOVA* Analysis of covariance, *Partial η*^*2*^ partial Eta Squared effect size

•Pairwise comparisons were performed using Bonferroni correction

••ANCOVA models were adjusted for pre-intervention stress and attachment scores

Regarding maternal attachment, attachment scores increased significantly after the intervention in the booklet-based education group (*p* = 0.004) and face-to-face education group (*p* = 0.008), whereas the increase in the control group was not statistically significant (*p* = 0.101). Nevertheless, no significant between-group differences were observed in post-intervention attachment scores (*F =* 0.96, *p* = 0.384) (Tables 3). Consistently, adjusted pairwise comparisons and ANCOVA revealed no significant intervention effect on maternal attachment after controlling for pre-intervention attachment scores (*F =* 0.94, *p* = 0.394, partial η^2^ = 0.021) (Tables 4).

## Discussion

The present study extends previous NICU parent education research by evaluating an APIB-based behavioral assessment educational intervention delivered through two modalities. Although both educational interventions were associated with improved outcomes, mothers who received education through booklet and face-to-face methods reported lower post-intervention stress levels than those in the control group after adjustment for pre-intervention values. The reduction in maternal stress observed in the control group may reflect the beneficial effects of routine NICU care and standard parental support. Nevertheless, structured APIB-based education demonstrated an added effect beyond routine care.

The beneficial effects observed in this study may be partly explained by the nature of the educational content. Unlike routine parent education, the intervention was based on APIB and Als’ synactive theory, which conceptualizes infant behavior as an organized interaction among autonomic, motor, state regulation, attention, and self-regulatory systems through which preterm infants communicate stress, stability, and readiness for interaction [8]. Improving mothers’ ability to recognize and interpret these behavioral cues may reduce ambiguity in understanding infant signals and enhance responsive caregiving [12, 36]. Consistent with this interpretation, Ramos et al. (2024) reported significant associations between maternal stress and lower neurobehavioral performance among preterm infants [37].

However, evidence regarding the effectiveness of educational interventions on parental stress in NICU settings remains inconsistent. A pilot randomized controlled trial found no significant improvement in maternal depressive symptoms after mothers of very low birth weight infants participated in a neonatal behavioral assessment scale (NBAS) intervention [38]. The authors attributed this finding partly to limited statistical power. Similarly, systematic reviews have reported variable effects of parent-focused educational interventions on stress outcomes, suggesting that information provision alone may be insufficient to improve parental psychological well-being [39, 40]. The content, delivery, and supportive components of educational interventions may therefore play an important role in determining effectiveness.

Unlike maternal stress, maternal attachment showed no significant between-group differences after adjustment for pre-intervention scores, despite improvements within the intervention group. Maternal attachment is a complex psychological construct that develops gradually through ongoing emotional interactions and caregiving experiences and may therefore be less responsive to short-term educational interventions [14, 41]. The relatively short follow-up period in the present study may also have limited the ability to detect between-group differences, as meaningful changes in maternal attachment are likely to emerge over a longer period. Therefore, the absence of significant between-group differences should not be interpreted as evidence of a lack of intervention efficacy. Previous reviews have similarly reported that interventions incorporating guided parent–infant interaction, emotional support, and confidence-building strategies produce greater improvements in parental outcomes than educational interventions focused primarily on information provision [42, 43]. These findings suggest that enhancing maternal attachment may require sustained opportunities for mother–infant interaction in addition to educational support.

Regarding educational intervention, previous studies comparing different approaches in NICU populations have generally shown beneficial effects of both face-to-face and alternative educational methods on parental outcomes, including anxiety, attachment, knowledge, and self-efficacy [29, 44–46]. In the present study, however, booklet-based education showed numerically greater effects than face-to-face education. This difference may be attributable to opportunities for repeated review of educational materials, self-paced learning, and reinforcement of knowledge over time. Mothers may have revisited booklet content while observing their infants’ behaviors in the NICU environment, thereby facilitating continued interpretation and application of behavioral cues. Nevertheless, the findings suggest that the educational content itself, namely improving mothers’ interpretation of preterm infant behavioral cues, may be more influential than the delivery modality.

### Clinical implications

The findings of this study suggest that integrating APIB-based behavioral assessment education into routine NICU parent support programs may provide a practical approach to supporting mothers of preterm infants during hospitalization. Educational interventions focused on recognizing and interpreting infant behavioral cues may help mothers better understand their infants’ needs and adapt to the NICU environment. Given the relatively simple implementation and low resource requirements of booklet-based education, such approaches may be particularly useful in busy neonatal units or settings with limited opportunities for prolonged face-to-face education. Incorporating structured behavioral education into routine parental support may contribute to more family-centered neonatal care by reducing maternal stress.

### Strengths and limitations

This study has several strengths. To our knowledge, limited evidence has specifically examined APIB-based behavioral assessment education among mothers of preterm infants in NICU settings. The use of standardized educational content delivered through two educational approaches alongside a control group enabled differentiation between the effects of educational delivery and content. In addition, outcome assessment by a blinded assessor and adjustment for pre-intervention scores strengthened the methodological rigor of the study. Several limitations should also be acknowledged. The quasi-experimental design and sequential (non-randomized) recruitment of the study groups, which was adopted to minimize information contamination between participants, may have reduced the study's internal validity despite efforts to control for potential confounding variables. Because the intervention and control groups were enrolled at different time points, unmeasured temporal changes—such as differences in clinical practice, environmental conditions, or participant characteristics—may have influenced the observed outcomes, introducing the possibility of temporal bias. The study was conducted in a single center, which may restrict generalizability. Furthermore, outcomes were assessed shortly after intervention completion; therefore, the long-term effects of APIB-based education, particularly on maternal attachment and mother–infant relationships, remain unclear. Future studies with extended follow-up periods are recommended to evaluate the sustainability of these effects over time.

## Conclusion

APIB-based behavioral assessment education may represent a feasible approach for supporting mothers of preterm infants in NICU settings. Educational interventions aimed at improving mothers’ interpretation of infant behavioral cues were associated with reduced maternal stress, whereas effects on maternal attachment appeared less pronounced in the short term. Although booklet-based education showed slightly greater effectiveness than face-to-face education, the findings suggest that the educational content itself, namely enhancing mothers’ understanding of preterm infant behavior, may be more influential than the method of education.

## Data Availability

All data generated or analyzed during this study are included in this published article and its supplementary information files.

## Abbreviations

NICUs: Neonatal Intensive Care Units
APIB: Assessment of Preterm Infants’ Behavior
TREND: Transparent Reporting of Evaluations with Nonrandomized Designs
PSS: Parental Stress Scale
CVR: Content Validity Ratio
CVI: Content Validity Index
MAI: Maternal Attachment Inventory
SD: Standard Deviation
ANOVA: Analysis of Variance
ANCOVA: Analysis of Covariance
NBAS: Neonatal Behavioral Assessment Scale
